# Supplementary material for: Bullying victimization and child sexual abuse among left-behind and non-left-behind children in China
Source: PeerJ. 2018 Jun 4;6:e4865. doi: 10.7717/peerj.4865 (PMC5991295; doi:10.7717/peerj.4865)
Supplement: Table S1 [file peerj-06-4865-s001.docx]

**eTable 1** Adjusted associations between bullying victimization and CSA

|  | Total | LBC | Non-LBC |
| --- | --- | --- | --- |
|  | OR (95%CI, *p* value) | OR(95%CI, *p* value) | OR(95%CI, *p* value) |
| Bullying victimization | 2.35(1.68-3.30, <0.001) | 2.52(1.34-4.73,0.004) | 2.35(1.58,3.53, <0.001) |
| Gender |  |  |  |
| Girls vs Boys | 0.20(0.14-0.29, <0.001) | 0.23(0.12-0.44, <0.001) | 0.18(0.11-0.28, <0.001) |
| Age (years) |  |  |  |
| 16-18 vs 11-15 | 1.56(1.11-2.20,0.010) | 1.72(0.91-3.25,0.096) | 1.57(1.03-2.37,0.033) |
| Home place |  |  |  |
| Rural vs Urban | 1.01(0.89-1.15, 0.897) | 0.93(0.72-1.21,0.586) | 1.01(0.87-1.17, 0.927) |
| Only child |  |  |  |
| No vs Yes | 1.04(0.73-1.48, 0.818) | 0.84(0.45-1.58,0.592) | 1.19(0.78-1.83,0.421) |
| Family structure |  |  |  |
| Non-traditional vs Traditional | 0.70(0.40-1.21,0.201) | 0.52(0.21-1.30,0.160) | 0.82(0.40-1.66,0.576) |
| Relationship with mother |  |  |  |
| Fine vs good | 1.60(0.92-2.78,0.096) | 1.31(0.53-3.23,0.559) | 1.74(0.85-3.57,0.132) |
| General vs good | 0.78(0.29-2.13,0.635) | 0.78(0.14-4.36,0.780) | 0.77(0.22-2.69,0.681) |
| Relationship with father |  |  |  |
| Fine vs good | 1.40(0.88-2.22,0.160) | 1.26(0.53-3.02,0.602) | 1.43(0.82-2.49,0.212) |
| General vs good | 1.39(0.62-3.11,0.419) | 1.06(0.311-3.64,0.921) | 1.57(0.52-4.79,0.426) |
| Parental educational level |  |  |  |
| General vs low | 1.10(0.70-1.73,0.673) | 0.81(0.34-1.91,0.629) | 1.25(0.73-2.14,0.418) |
| High vs low | 0.85(0.39-1.87,0.695) | 1.23(0.21-7.33,0.822) | 0.86(0.35-2.08,0.731) |

*Adjusted potential confounders, including age, gender, home place, only child, family structure, relationship with mother, relationship with father, parental educational level.
