# Supplementary material for: Bullying victimization and child sexual abuse among left-behind and non-left-behind children in China
Source: PeerJ. 2018 Jun 4;6:e4865. doi: 10.7717/peerj.4865 (PMC5991295; doi:10.7717/peerj.4865)
Supplement: Table S2 [file peerj-06-4865-s002.docx]

**eTable 2** Adjusted associations between bullying victimization and CSA in boys

|  | Total | LBC | Non-LBC |
| --- | --- | --- | --- |
|  | OR (95%CI, *p* value) | OR(95%CI, *p* value) | OR(95%CI, *p* value) |
| Bullying victimization | 2.02(1.34-3.03,0.001) | 1.32(0.58-2.98,0.501) | 2.34(1.44-3.79,0.001) |
| Age (years) |  |  |  |
| 16-18 vs 11-15 | 2.02(1.34-3.03,0.001) | 2.16(0.95-4.92,0.067) | 2.05(1.27-3.34,0.004) |
| Home place |  |  |  |
| Rural vs Urban | 1.04(0.89-1.21,0.639) | 1.06(0.76-1.47,0.751) | 1.02(0.86-1.22, 0.816) |
| Only child |  |  |  |
| No vs Yes | 1.07(0.71-1.63,0.733) | 1.27(0.57-2.83,0.552) | 1.09(0.66-1.80,0.728) |
| Family structure |  |  |  |
| Non-traditional vs Traditional | 0.71(0.36-1.39,0.318) | 0.41(0.12-1.38,0.152) | 0.91(0.40-2.05,0.811) |
| Relationship with mother |  |  |  |
| Fine vs good | 1.71(0.87-3.37,0.122) | 2.24(0.68-7.42,0.185) | 1.55(0.65-3.70,0.324) |
| General vs good | 0.93(0.29-2.95,0.903) | 3.80(0.40-36.47,0.247) | 0.62(0.14-2.82,0.540) |
| Relationship with father |  |  |  |
| Fine vs good | 1.22(0.69-2.18,0.493) | 1.07(0.30-3.84,0.914) | 1.30(0.67-2.52,0.429) |
| General vs good | 1.38(0.53-3.63,0.510) | 1.22(0.30-4.89,0.780) | 1.62(0.36-7.36,0.531) |
| Parental educational level |  |  |  |
| General vs low | 1.02(0.59-1.76,0.950) | 0.92(0.30-2.79,0.887) | 1.03(0.54-1.96,0.922) |
| High vs low | 1.00(0.41-2.40,0.994) | 2.22(0.29-17.07,0.442) | 0.87(0.32-2.35,0.781) |

*Adjusted potential confounders, including age, home place, only child, family structure, relationship with mother, relationship with father, parental educational level.
