# Supplementary material for: Bullying victimization and child sexual abuse among left-behind and non-left-behind children in China
Source: PeerJ. 2018 Jun 4;6:e4865. doi: 10.7717/peerj.4865 (PMC5991295; doi:10.7717/peerj.4865)
Supplement: Table S3 [file peerj-06-4865-s003.docx]

**eTable 3** Adjusted associations between bullying victimization and CSA in girls

|  | Total | LBC | Non-LBC |
| --- | --- | --- | --- |
|  | OR (95%CI, *p* value) | OR(95%CI, *p* value) | OR(95%CI, *p* value) |
| Bullying victimization | 3.40(1.81-6.38, <0.001) | 7.36(2.16-24.99,0.001) | 2.38(1.08-5.27,0.032) |
| Age (years) |  |  |  |
| 16-18 vs 11-15 | 0.88(0.47-1.63,0.678) | 1.54(0.48-4.98,0.462) | 0.71(0.32-1.55,0.386) |
| Home place |  |  |  |
| Rural vs Urban | 0.97(0.76-1.23,0.791) | 0.80(0.49-1.32,0.387) | 0.96(0.71-1.30, 0.784) |
| Only child |  |  |  |
| No vs Yes | 1.01(0.52-1.96,0.983) | 0.57(0.18-1.79,0.338) | 1.68(0.67-4.21,0.272) |
| Family structure |  |  |  |
| Non-traditional vs Traditional | 0.60(0.21-1.71,0.341) | 0.47(0.09-2.34,0.358) | 0.53(0.11-2.62,0.438) |
| Relationship with mother |  |  |  |
| Fine vs good | 1.36(0.51-3.64,0.544) | 0.50(0.08-3.01,0.448) | 2.00(0.56-7.19,0.289) |
| General vs good | 0.58(0.07-4.89,0.617) | ---- | 1.66(0.16-17.09,0.670) |
| Relationship with father |  |  |  |
| Fine vs good | 1.71(0.79-3.73,0.175) | 1.61(0.43-5.99,0.475) | 1.86(0.66-5.31,0.243) |
| General vs good | 1.06(0.21-5.26,0.942) | ---- | 2.07(0.36-11.99,0.414) |
| Parental educational level |  |  |  |
| General vs low | 1.47(0.67-3.23,0.332) | 0.62(0.13-2.94,0.551) | 2.45(0.93-6.47,0.071) |
| High vs low | 0.45(0.06-3.63,0.452) | ---- | 0.63(0.07-5.87,0.684) |

*Adjusted potential confounders, including age, home place, only child, family structure, relationship with mother, relationship with father, parental educational level.
