# Supplementary material for: Bullying victimization and child sexual abuse among left-behind and non-left-behind children in China
Source: PeerJ. 2018 Jun 4;6:e4865. doi: 10.7717/peerj.4865 (PMC5991295; doi:10.7717/peerj.4865)
Supplement: Table S4 [file peerj-06-4865-s004.docx]

**eTable 4** Adjusted associations between bullying victimization and CSA in children age 11-15

|  | Total | LBC | Non-LBC |
| --- | --- | --- | --- |
|  | OR (95%CI, *p* value) | OR(95%CI, *p* value) | OR(95%CI, *p* value) |
| Bullying victimization | 3.16(1.89-5.30, <0.001) | 3.42(1.18-9.93,0.023) | 3.32(1.80-6.15, <0.001) |
| Gender |  |  |  |
| Girls vs Boys | 0.32(0.19-0.55, <0.001) | 0.30(0.11-0.81,0.017) | 0.33(0.17-0.63,0.001) |
| Home place |  |  |  |
| Rural vs Urban | 1.03(0.85-1.24,0.772) | 0.93(0.64-1.37,0.730) | 1.04(0.83-1.29, 0.761) |
| Only child |  |  |  |
| No vs Yes | 0.94(0.56-1.57,0.802) | 0.63(0.23-1.75,0.376) | 1.10(0.58-2.08,0.770) |
| Family structure |  |  |  |
| Non-traditional vs Traditional | 1.03(0.53-2.02,0.921) | 0.97(0.33-2.94,0.969) | 1.11(0.46-2.70,0.813) |
| Relationship with mother |  |  |  |
| Fine vs good | 2.57(1.23-5.35,0.012) | 1.97(0.56-6.99,0.291) | 3.04(1.17-7.88,0.022) |
| General vs good | 1.27(0.41-3.98,0.680) | 1.12(0.16-7.69,0.907) | 1.58(0.35-7.24,0.554) |
| Relationship with father |  |  |  |
| Fine vs good | 1.50(0.78-2.91,0.220) | 1.04(0.27-4.02,0.950) | 1.54(0.70-3.37,0.283) |
| General vs good | 0.53(0.14-2.05,0.356) | 0.25(0.02-3.73,0.317) | 0.50(0.09-2.83,0.434) |
| Parental educational level |  |  |  |
| General vs low | 1.64 (0.87-3.11,0.128) | 1.18(0.31-4.50,0.808) | 1.83(0.87-3.87,0.112) |
| High vs low | 1.04(0.35-3.09,0.949) | 3.51(0.37-33.61,0.276) | 0.75(0.19-2.95,0.681) |

*Adjusted potential confounders, including gender, home place, only child, family structure, relationship with mother, relationship with father, parental educational level.
