# Supplementary material for: Bullying victimization and child sexual abuse among left-behind and non-left-behind children in China
Source: PeerJ. 2018 Jun 4;6:e4865. doi: 10.7717/peerj.4865 (PMC5991295; doi:10.7717/peerj.4865)
Supplement: Table S5 [file peerj-06-4865-s005.docx]

**eTable 5** Adjusted associations between bullying victimization and CSA in children age 16-18

|  | Total | LBC | Non-LBC |
| --- | --- | --- | --- |
|  | OR (95%CI, *p* value) | OR(95%CI, *p* value) | OR(95%CI, *p* value) |
| Bullying victimization | 1.78(1.10-2.87,0.018) | 2.06(0.86-4.91,0.105) | 1.65(0.92-2.97,0.093) |
| Gender |  |  |  |
| Girls vs Boys | 0.13(0.08-0.22, <0.001) | 0.19(0.08-0.47, <0.001) | 0.10(0.05-0.19, <0.001) |
| Home place |  |  |  |
| Rural vs Urban | 1.02(0.85-1.23,0.814) | 0.98(0.65-1.47,0.924) | 1.00(0.81-1.24, 0.995) |
| Only child |  |  |  |
| No vs Yes | 1.20(0.74-1.98,0.454) | 0.90(0.37-2.13,0.804) | 1.41(0.77-2.59,0.269) |
| Family structure |  |  |  |
| Non-traditional vs Traditional | 0.24(0.07-0.79,0.019) | 0.08(0.01-0.82,0.033) | 0.42(0.11-1.62,0.209) |
| Relationship with mother |  |  |  |
| Fine vs good | 0.99(0.41-2.38,0.978) | 0.91(0.23-3.62,0.899) | 0.92(0.29-2.96,0.889) |
| General vs good | 0.23(0.02-2.44,0.223) |  | 0.13(0.01-1.99,0.143) |
| Relationship with father |  |  |  |
| Fine vs good | 1.37(0.70-2.70,0.359) | 1.33(0.39-4.49,0.650) | 1.39(0.61-3.16,0.438) |
| General vs good | 3.79(1.15-12.52,0.029) | 2.72(0.43-17.22,0.288) | 7.68(1.40-42.08,0.019) |
| Parental educational level |  |  |  |
| General vs low | 0.83(0.43-1.59,0.567) | 0.73(0.22-2.38,0.601) | 0.88(0.39-1.98,0.759) |
| High vs low | 0.84(0.27-2.63,0.759) | -- | 0.98(0.29-3.29,0.979) |

* Adjusted potential confounders, including gender, home place, only child, family structure, relationship with mother, relationship with father, parental educational level.
