# Supplementary material for: Bullying victimization and child sexual abuse among left-behind and non-left-behind children in China
Source: PeerJ. 2018 Jun 4;6:e4865. doi: 10.7717/peerj.4865 (PMC5991295; doi:10.7717/peerj.4865)
Supplement: Table S6 [file peerj-06-4865-s006.docx]

**eTable 6** Adjusted associations between bullying victimization and CSA in urban children

|  | Total | LBC | Non-LBC |
| --- | --- | --- | --- |
|  | OR (95%CI, *p* value) | OR(95%CI, *p* value) | OR(95%CI, *p* value) |
| Bullying victimization | 1.55(0.85-2.82,0.157) | 0.96(0.23-4.06,0.956) | 1.65(0.83-3.30,0.153) |
| Gender |  |  |  |
| Girls vs Boys | 0.23(0.12-0.43, <0.001) | 0.34(0.08-1.36,0.128) | 0.17(0.08-0.38, <0.001) |
| Age (years) |  |  |  |
| 16-18 vs 11-15 | 1.29(0.71-2.37, 0.404) | 1.01(0.23-4.37,0.986) | 1.43(0.71-2.88, 0.312) |
| Only child |  |  |  |
| No vs Yes | 1.10(0.58-2.11,0.770) | 2.10(0.49-8.92,0.315) | 1.09(0.51-2.33,0.824) |
| Family structure |  |  |  |
| Non-traditional vs Traditional | 0.77(0.30-2.00,0.598) | 1.22(0.18-8.31,0.837) | 0.78(0.25-2.49,0.676) |
| Relationship with mother |  |  |  |
| Fine vs good | 2.43(0.95-6.22,0.065) | 3.93(0.61-25.20,0.149) | 1.63(0.48-5.56,0.434) |
| General vs good | 0.27(0.03-2.36,0.239) |  | 0.23(0.02-2.51,0.230) |
| Relationship with father |  |  |  |
| Fine vs good | 1.23(0.58-2.64,0.584) | 0.54(0.08-3.53,0.521) | 1.44(0.60-3.47,0.416) |
| General vs good | 1.50(0.40-5.60,0.543) | 0.32(0.02-4.73,0.410) | 3.71(0.62-22.21,0.151) |
| Parental educational level |  |  |  |
| General vs low | 0.87(0.44-1.74,0.699) | 0.79(0.20-3.07,0.731) | 0.88(0.38-2.01,0.755) |
| High vs low | 0.92(0.38-2.25,0.864) | 1.34(0.05-39.14,0.863) | 1.02(0.39-2.66,0.973) |

* Adjusted potential confounders, including gender, age, only child, family structure, relationship with mother, relationship with father, parental educational level.
