# Supplementary material for: Bullying victimization and child sexual abuse among left-behind and non-left-behind children in China
Source: PeerJ. 2018 Jun 4;6:e4865. doi: 10.7717/peerj.4865 (PMC5991295; doi:10.7717/peerj.4865)
Supplement: Table S7 [file peerj-06-4865-s007.docx]

**eTable 7** Adjusted associations between bullying victimization and CSA in rural children

|  | Total | LBC | Non-LBC |
| --- | --- | --- | --- |
|  | OR (95%CI, *p* value) | OR(95%CI, *p* value) | OR(95%CI, *p* value) |
| Bullying victimization | 2.82(1.86-4.26, <0.001) | 2.97(1.42-6.22,0.004) | 2.92(1.74-4.89, <0.001) |
| Gender |  |  |  |
| Girls vs Boys | 0.18(0.11-0.28, <0.001) | 0.18(0.08-0.39, <0.001) | 0.17(0.10-0.30, <0.001) |
| Age (years) |  |  |  |
| 16-18 vs 11-15 | 1.70(1.12-2.60, 0.013) | 1.90(0.89-4.07,0.098) | 1.62(0.96-2.73, 0.071) |
| Only child* |  |  |  |
| No vs Yes | 0.97(0.63-1.47,0.873) | 0.70(0.34-1.43,0.324) | 1.21(0.71-2.07,0.491) |
| Family structure |  |  |  |
| Non-traditional vs Traditional | 0.63(0.31-1.28,0.205) | 0.44(0.14-1.36,0.155) | 0.85(0.34-2.12,0.720) |
| Relationship with mother |  |  |  |
| Fine vs good | 1.35(0.67-2.73,0.407) | 0.82(0.25-2.62,0.734) | 1.94(0.78-4.83,0.157) |
| General vs good | 1.26(0.39-4.07,0.698) | 1.10(0.18-6.85,0.918) | 1.57(0.33-7.53,0.572) |
| Relationship with father |  |  |  |
| Fine vs good | 1.51(0.83-2.74,0.181) | 1.60(0.54-4.72,0.398) | 1.41(0.67-2.97,0.363) |
| General vs good | 1.26(0.45-3.52,0.657) | 1.60(0.34-7.54,0.552) | 0.89(0.20-4.01,0.875) |
| Parental educational level |  |  |  |
| General vs low | 1.32(0.73-2.41,0.348) | 0.79(0.24-2.57,0.700) | 1.69(0.84-3.42,0.144) |
| High vs low | 0.34(0.04-2.90,0.322) | 1.23(0.11-13.84,0.870) | --- |

* Adjusted potential confounders, including gender, age, only child, family structure, relationship with mother, relationship with father, parental educational level.
