# Supplementary material for: Bullying victimization and child sexual abuse among left-behind and non-left-behind children in China
Source: PeerJ. 2018 Jun 4;6:e4865. doi: 10.7717/peerj.4865 (PMC5991295; doi:10.7717/peerj.4865)
Supplement: Table S9 [file peerj-06-4865-s009.docx]

**eTable 9** Adjusted associations between bullying victimization and CSA in non-only children

|  | Total | LBC | Non-LBC |
| --- | --- | --- | --- |
|  | OR (95%CI, *p* value) | OR(95%CI, *p* value) | OR(95%CI, *p* value) |
| Bullying victimization | 2.81(1.72-4.57, <0.001) | 3.85(1.37-10.85,0.011) | 2.58(1.46-4.54,0.001) |
| Gender |  |  |  |
| Girls vs Boys | 0.18(0.11-0.30, <0.001) | 0.15(0.05-0.42, <0.001) | 0.20(0.11-0.36, <0.001) |
| Age (years) |  |  |  |
| 16-18 vs 11-15 | 1.89(1.15-3.10, 0.011) | 2.49(0.83-7.49,0.105) | 1.71(0.96-3.03, 0.066) |
| Home place |  |  |  |
| Rural vs Urban | 0.96(0.78-1.17,0.673) | 0.83(0.52-1.31,0.423) | 1.00(0.79-1.26,0.980) |
| Family structure |  |  |  |
| Non-traditional vs Traditional | 1.00(0.39-2.59,0.994) | 0.84(0.16-4.44,0.837) | 1.25(0.38-4.12,0.718) |
| Relationship with mother |  |  |  |
| Fine vs good | 1.72(0.74-4.00,0.207) | 1.20(0.27-5.41,0.815) | 2.29(0.78-6.68,0.130) |
| General vs good | 0.65(0.155-2.73,0.557) | 0.57(0.03-11.57,0.717) | 0.71(0.13-3.88,0.695) |
| Relationship with father |  |  |  |
| Fine vs good | 2.15(1.13-4.12,0.020) | 2.11(0.55-8.08,0.276) | 2.15(1.01-4.62,0.047) |
| General vs good | 1.77(0.48-6.50,0.391) | 2.70(0.22-32.61,0.435) | 0.97(0.16-5.95,0.975) |
| Parental educational level |  |  |  |
| General vs low | 1.71(0.90-3.27,0.103) | 2.04(0.58-7.19,0.266) | 1.57(0.72-3.42,0.260) |
| High vs low | 0.23(0.02-2.42,0.222) | --- | 0.25(0.02-2.87,0.265) |

* Adjusted potential confounders, including gender, age, home place, family structure, relationship with mother, relationship with father, parental educational level.
