# Supplementary material for: Bullying victimization and child sexual abuse among left-behind and non-left-behind children in China
Source: PeerJ. 2018 Jun 4;6:e4865. doi: 10.7717/peerj.4865 (PMC5991295; doi:10.7717/peerj.4865)
Supplement: Table S10 [file peerj-06-4865-s010.docx]

**eTable 10** Adjusted associations between bullying victimization and CSA in children with traditional family structure

|  | Total | LBC | Non-LBC |
| --- | --- | --- | --- |
|  | OR (95%CI, *p* value) | OR(95%CI, *p* value) | OR(95%CI, *p* value) |
| Bullying victimization | 2.20(1.54-3.14, <0.001) | 2.69(1.35-5.34,0.005) | 2.13(1.40-3.26, <0.001) |
| Gender |  |  |  |
| Girls vs Boys | 0.19(0.13-0.29, <0.001) | 0.22(0.11-0.44, <0.001) | 0.18(0.11-0.30, <0.001) |
| Age (years) |  |  |  |
| 16-18 vs 11-15 | 1.77(1.23-2.53,0.002) | 2.21(1.10-4.44,0.026) | 1.66(1.08-2.56,0.022) |
| Home place |  |  |  |
| Rural vs Urban | 1.01(0.89-1.16,0.834) | 0.92(0.69-1.22,0.556) | 1.01(0.86-1.18,0.906) |
| Only child |  |  |  |
| No vs Yes | 1.00(0.69-1.44, 0.987) | 0.77(0.39-1.51,0.450) | 1.13(0.73-1.77,0.584) |
| Relationship with mother |  |  |  |
| Fine vs good | 1.48(0.80-2.73,0.211) | 0.86(0.30-2.49,0.781) | 1.93(0.90-4.16,0.093) |
| General vs good | 0.88(0.28-2.71,0.818) | 0.46(0.04-5.03,0.528) | 1.14(0.32-4.14,0.839) |
| Relationship with father |  |  |  |
| Fine vs good | 1.37(0.84-2.26,0.211) | 1.01(0.38-2.71,0.985) | 1.50(0.84-2.69,0.170) |
| General vs good | 1.69(0.67-4.31,0.269) | 1.73(0.37-8.11,0.490) | 1.46(0.42-5.07,0.548) |
| Parental educational level |  |  |  |
| General vs low | 1.10(0.69-1.75,0.696) | 0.80(0.31-2.04,0.640) | 1.22(0.71-2.12,0.468) |
| High vs low | 0.71(0.29-1.76,0.459) | 0.73(0.07-7.84,0.795) | 0.74(0.27-2.02,0.562) |

* Adjusted potential confounders, including age, gender, home place, only child, relationship with mother, relationship with father, parental educational level.
