# Supplementary material for: Bullying victimization and child sexual abuse among left-behind and non-left-behind children in China
Source: PeerJ. 2018 Jun 4;6:e4865. doi: 10.7717/peerj.4865 (PMC5991295; doi:10.7717/peerj.4865)
Supplement: Table S11 [file peerj-06-4865-s011.docx]

**eTable 11** Adjusted associations between bullying victimization and CSA in children with non-traditional family structure

|  | Total | LBC | Non-LBC |
| --- | --- | --- | --- |
|  | OR (95%CI, *p* value) | OR(95%CI, *p* value) | OR(95%CI, *p* value) |
| Bullying victimization | 5.95(1.46-24.21,0.013) | 11.72(0.36-379.20,0.165) | 12.25(1.51-99.63,0.019) |
| Gender |  |  |  |
| Girls vs Boys | 0.25(0.07-086, 0.028) | 0.57(0.04-8.39,0.684) | 0.07(0.01-0.72, 0.025) |
| Age (years) |  |  |  |
| 16-18 vs 11-15 | 0.52(0.14-1.97,0.333) | 0.11(0.00-3.70, 0.222) | 1.32(0.19-9.01,0.776) |
| Home place |  |  |  |
| Rural vs Urban | 1.01(0.64-1.60,0.973) | 1.23(0.31-4.87,0.765) | 1.07(0.56-2.03,0.844) |
| Only child |  |  |  |
| No vs Yes | 2.35(0.58-9.51, 0.232) | 1.90(0.09-39.44,0.678) | 2.91(0.33-25.61,0.336) |
| Relationship with mother |  |  |  |
| Fine vs good | 3.45(0.74-16.17,0.116) | 18.16(0.47-701.81,0.120) | 0.89(0.07-11.28,0.927) |
| General vs good | 0.78(0.08-7.94,0.837) | 5.05(0.12-212.93,0.396) | --- |
| Relationship with father |  |  |  |
| Fine vs good | 1.90(0.44-8.26,0.394) | 5.40(0.11-262.14,0.395) | 0.96(0.07-14.17,0.977) |
| General vs good | 0.96(0.15-6.04,0.962) | 4.94(0.10-249.77,0.425) | 3.80(0.16-90.44,0.409) |
| Parental educational level |  |  |  |
| General vs low | 1.14(0.17-7.72,0.890) | 0.65(0.03-12.46,0.777) | 4.55(0.18-113.07,0.355) |
| High vs low | 2.75(0.42-18.17,0.293) | 1.72(0.02-163.76,0.816) | 3.23(0.25-40.95,0.366) |

* Adjusted potential confounders, including age, gender, home place, only child, relationship with mother, relationship with father, parental educational level.
