# Supplementary material for: Bullying victimization and child sexual abuse among left-behind and non-left-behind children in China
Source: PeerJ. 2018 Jun 4;6:e4865. doi: 10.7717/peerj.4865 (PMC5991295; doi:10.7717/peerj.4865)
Supplement: Supplemental Information 2 [file peerj-06-4865-s013.docx]

**欺凌与儿童性虐待问卷**

**Bullying Victimization and Child Sexual Abuse**

**背景信息（Background information）**

1.请选择你所在的省市：1. 安徽 2. 四川

Province 1.Anhui 2. Sichuan

2.你的出生年月日

Date of birth: ____year ____month ____day

3.请选择你的性别 1.男孩 2.女孩

Sex: 1. Boy 2. Girl

4.你现在是几年级？ 1. 初一 2. 初二 3.初三 4.高一 5.高二 6.高三

What grade are you in now? 1. Grade 7 2. Grade 8 3.Grade 9 4.Grade10 5.Grade11 6.Grade 12

5.你的家庭所在地是 1.城市2.农村

Which type of home place do you belong to? 1. Urban area 2. Rural area

6.你是否为独生子女？ 1.是 2.不是

Do you have siblings ? 1.Yes 2.No

7.你亲生父母的婚姻状况如何？

1.正常2.亲生父母离婚了3.亲生母亲去世了4.亲生父亲去世了5.亲生父母都去世了

What are your biological parents' marital status?

1. Married 2. Divoce 3. Mother passed away 4. Father passed away 5.Both parents passed away

8.你现在母亲（亲生母亲或养母、继母）的文化程度是

1.初中以下 2.高中/中专/技校 3.大专或大专以上 4.不知道 5.没有母亲

What is the highest degree of education your mother (Biological mother, foster mother, or stepmother) obtained?

1. Junior high school or below 2. High school/ vocational school 3. College or above

4. I don’t know 5. I have no mother

9.你母亲最近是否一直在外地打工？（在外打工期间，中间偶尔有事临时回家或回家过节计为在外打工。）

1.否，最近半年一直没有外出打工 2.是，但最近半年外出打工不满6个月

3.是，最近半年一直在外打工 4.是，最近一年一直在外打工

Has your mother been working outside recently?

1. No 2. Yes, but less than 6 months 3. Yes, 6 months 4. Yes, 12 months

10.你觉得你与你母亲的关系属于 1.非常好 2.较好 3.一般 4.不太好 5.很不好

How is the relationship between you and your mother? 1. Good 2. General 3.Poor

11.你现在父亲（亲生父亲或养父、继父）的文化程度是

1.初中以下 2.高中/中专/技校 3.大专或大专以上 4.不知道 5.没有父亲

What is the highest degree of education your (Biological father, foster father, or stepfather) obtained?

1.Junior high school or below 2. High school/vocational school 3. College or above

4.I don’t know 5.I have no father

12.你父亲最近是否一直在外地打工？（在外打工期间，中间偶尔有事临时回家或回家过节计为在外打工。）

1.否，最近半年一直没有外出打工 2.是，但最近半年外出打工不满6个月

3.是，最近半年一直在外打工 4.是，最近一年一直在外打工

Has your father been working outside recently?

1. No 2. Yes, but less than 6 months 3. Yes, 6 months 4. Yes, 12 months

13.你觉得你与你父亲的关系

1.非常好 2.较好 3.一般 4.不太好 5.很不好

How is the relationship between you and your father? 1. Good 2. General 3.Poor

**欺凌问卷（Bullying victimization）**

14.在过去的一年里，你有几次被同学用刀、棍等武器威胁或伤害过？

1.0次 2.1次 3.2-3次 4.4-5次 5.6-8次 6.9-11次 7.12次及以上

During the past year, I was threatened or hurt by my classmates with swords, clubs and other weapons.

0=never 1=1-2times/year 2=3-5times/year 3=6-11times/year 4=1-2times/month 5=1-2times/week 6 ≥ 2 times/week

15.在过去的一年里，你是否单独被体力明显比你强的一个或几个同学打、踢、推、关在房间内？

1.从没有 2.1-2次 3.3-5次 4.6-11次 5.每月1-2次 6.每周1-2次 7.每周大于2次

During the past year, I was hit, kicked, pushed, shoved around, or locked indoors.

0=never 1=1-2times/year 2=3-5times/year 3=6-11times/year 4=1-2times/month 5=1-2times/week 6 ≥ 2 times/week

16.在过去的一年里，你是否单独被体力明显比你强的一个或几个同学以伤害性方式取笑或戏弄过？

1.从没有 2.1-2次 3.3-5次 4.6-11次 5.每月1-2次 6.每周1-2次 7.每周大于2次

During the past year, I was called mean names, was made fun of, or teased in a hurtful way.

0=never 1=1-2times/year 2=3-5times/year 3=6-11times/year 4=1-2times/month 5=1-2times/week 6 ≥ 2 times/week

17.在过去的一年里，你是否单独被体力明显比你强的一个或几个同学不友好的取过绰号？

1.从没有 2.1-2次 3.3-5次 4.6-11次 5.每月1-2次 6.每周1-2次 7.每周大于2次

During the past year, other students made sexual jokes, comments, or gestures to me.

0=never 1=1-2times/year 2=3-5times/year 3=6-11times/year 4=1-2times/month 5=1-2times/week 6 ≥ 2 times/week

18.在过去的一年里，你是否单独被体力明显比你强的一个或几个同学孤立在一群朋友同学外或被有意忽略？

1.从没有 2.1-2次 3.3-5次 4.6-11次 5.每月1-2次 6.每周1-2次 7.每周大于2次

During the past year, other students left me out of things on purpose, excluded me from their group of friends, or completely ignored me.

0=never 1=1-2times/year 2=3-5times/year 3=6-11times/year 4=1-2times/month 5=1-2times/week 6 ≥ 2 times/week

19.过去的一年里，你是否单独被体力明显比你强的一个或几个同学欺骗或传过关于你的谣言或绯闻？

1.从没有 2.1-2次 3.3-5次 4.6-11次 5.每月1-2次 6.每周1-2次 7.每周大于2次

During the past year, other students told lies or spread false rumors about me and tried to make others dislike me.

0=never 1=1-2times/year 2=3-5times/year 3=6-11times/year 4=1-2times/month 5=1-2times/week 6 ≥ 2 times/week

20.过去的一年里，你是否单独被体力明显比你强的一个或几个同学通过手机电话、短信、QQ、微信等电子工具欺负过？

1.从没有 2.1-2次 3.3-5次 4.6-11次 5.每月1-2次 6.每周1-2次 7.每周大于2次

During the past year, I was bullied by my classmates using a phone, e-mail message or pictures.

0=never 1=1-2times/year 2=3-5times/year 3=6-11times/year 4=1-2times/month 5=1-2times/week 6 ≥ 2 times/week

**儿童性虐待（Child sexual abuse）**

21.是否有人让你观看色情书刊、电影、DVD、图片、录像、视频等？指年龄比你大的人。

1.是 2.否

Has an adult or an older child ever not respected you by demanding you or forcing you to look at pornographic pictures, drawings, films, videotapes or magazines? 1.Yes 2.No

22.是否有年龄比你大的人对你讲色情言论（包括黄段子、黄色笑话或故事）

或用色情言语辱骂你？ 1.是 2.否

Has an adult or an older child ever not respected you by sexually explicit talk or hostile language? 1. Yes 2.No

23.是否有年龄比你大的人把你的裸体绘成画或拍成照片或录像？ 1.是 2.否

Has an adult or an older child ever not respected you by demanding you or forcing you to be naked and to expose your genitals for picture taking or filming? 1. Yes 2.No

24.是否有年龄比你大的人在你面前暴露或玩弄其性器官？ 1.是 2.否

Has an adult or an older child ever not respected you by demanding you or forcing you to look at his/her genitals or watch him/her masturbate? 1. Yes 2.No

25.是否有年龄比你大的人窥探你的乳房或性器官？ 1.是 2.否

Has an adult or an older child ever peeked at your breast or genital? 1. Yes 2.No

26.是否有年龄比你大的人触摸你的乳房或性器官或用嘴接触你的性器官或故意在你身上摩擦其性器官？ 1.是 2.否

Has an adult or an older child ever not respected you by demanding you or forcing you to be fondled (caresses, rubs, kisses on the whole body and/or your genitals)? 1. Yes 2.No

27.是否有年龄比你大的人强迫你触摸或用嘴接触他/她的性器官？1.是 2.否

Has an adult or an older child ever not respected you by demanding you or forcing you to be fondled him/her (caresses, rubs, kisses on the whole body and/or his/her genitals)? 1. Yes 2.No

28.是否有年龄比你大的人在你的阴道中放置异物？ 1.是 2.否

Has an adult or an older child ever not respected you by demanding you or forcing you to submit to having his/her fingers or an object introduced in your body? 1. Yes 2.No

29.是否有年龄比你大的人试图（但未成功）将其生殖器插入你的肛门或阴道？1.是 2.否

Has an adult or an older child ever tried to making anal sex or vaginal sex with you?

1. Yes 2.No

30.是否有年龄比你大的人将其生殖器插入你的肛门或阴道？ 1.是 2.否

Has an adult or an older child ever made anal sex or vaginal sex with you? 1. Yes 2.No
